# Effects of historical inequity and institutional power on cannabis research: Moving toward equity and inclusion

**DOI:** 10.1093/pnasnexus/pgad383

**Published:** 2023-12-12

**Authors:** Renée Martin-Willett, Madeline Stanger, Wanda James, Angela D Bryan, L Cinnamon Bidwell

**Affiliations:** Department of Psychology and Neuroscience, University of Colorado Boulder, Boulder, CO 80309, USA; Department of Psychology and Neuroscience, University of Colorado Boulder, Boulder, CO 80309, USA; University of Colorado Board of Regents, 1st Congressional District, Denver, CO 80203, USA; Department of Psychology and Neuroscience, University of Colorado Boulder, Boulder, CO 80309, USA; Department of Psychology and Neuroscience, University of Colorado Boulder, Boulder, CO 80309, USA; Institute of Cognitive Science, University of Colorado Boulder, Boulder, CO 80309, USA

**Keywords:** cannabis, marijuana, underrepresented groups, equity, drug policy

## Abstract

Given historical inequities in cannabis laws and policies, there is an obligation on the part of researchers and policy makers to actively work toward improving equity in cannabis research at a time when the field is rapidly expanding. We wish to propose a way forward for cannabis research that acknowledges this history of discrimination and misuse of institutional power and embraces equity and inclusion. This article provides a brief perspective on historical drug policy, recent legalization trends that have disproportionately benefitted some groups over others, and the repercussions of those trends for the cannabis research enterprise. In addition, it proposes five key actions in both policy and research domains that are necessary to move the field of cannabis research, and perhaps biomedical research in substance use more broadly, forward in a productive and inclusionary way. Specifically, recommendations focus on equity-focused legislation and policy, supporting the entry and retention of scientists of color into the field, engaging in more ethical research practices, and practicing intentionally inclusive recruitment of participants will help to move the field of cannabis research forward. These efforts will ensure that scientific gains are shared equitably moving forward.

As early as 2700 BC, cannabis was in use as a textile as well as religiously, recreationally, and medicinally in diverse societies around the globe (1–3), including Western, Southern, and Eastern Asia (4) and in indigenous and Latin American cultures in North, Central, and South America (5, 6). At the same time, there has also been a long history of different societies using substance use laws and policies to exert power or control over specific groups (7).

The racialization of the so-called War on Drugs has been well documented across multiple literatures (8). In the case of cannabis, in particular, there is evidence that communities of color have been disproportionately prosecuted and incarcerated (9–13), even though epidemiological data suggest cannabis use rates between Hispanic, black, and white groups are comparable (14). As a result, existing distrust of the biomedical research establishment by communities of color (15, 16) may be exacerbated in the case of substance use research and cannabis research in particular.

Given these circumstances, we wish to propose a way forward for cannabis research that acknowledges this history of discrimination and misuse of institutional power and embraces equity and inclusion. First, we will provide a brief overview of the history of cannabis legal policy and enforcement in the United States. Then, we will discuss how recent legalization trends have disproportionately benefitted some groups over others and the repercussions of those trends for the cannabis research enterprise. Finally, we propose five key actions in both policy and research domains that are necessary to move the field of cannabis research, and perhaps biomedical research in substance use more broadly, forward in a productive and inclusionary way.

## History of cannabis legal policy and enforcement in the United States

Cannabis was mostly considered a safe recreational or medicinal substance in the United States until the 20th century (17, 18). Interestingly, while inconsistently banned on a state-by-state basis during Prohibition, cannabis was not banned from nonmedical use federally until five years after Prohibition ended (19).^1^ Despite the fact that few in the Hispanic community used cannabis (20), and many Mexican Americans viewed cannabis use as “a badge of inferiority” (10), white legislators in the Western and Southeastern United States used racialized language in their arguments for cannabis prohibition (19) that were in reality prompted by fears surrounding rising use by young white people (21).

Though it had been recognized by the President's Commission on Crime in 1967 that US drug policies were discriminatory and ineffective (22), legislators in the 1960s grew increasingly preoccupied with cannabis regulation, perhaps linked to the fact that young white adults in the middle and upper classes were using cannabis in large numbers (23). Circumstances reached a fever pitch in 1971 when marijuana was listed as a Schedule I drug (24). Since that time, millions of Americans have been prosecuted for marijuana-related offenses with striking racial disparities in arrest rates (25). Now, even though recreational cannabis use is legal in 37 states and the District of Columbia, there were still over 170,000 marijuana possession-related arrests in the United States in 2021 that continued to disproportionately target communities of color (26).

Being found guilty of the federal offense of simple possession of marijuana has numerous legal implications that continue to affect individuals for years, even as states and the federal government pursue marijuana offense-related pardoning (27, 28). Offenders receive criminal “history” points, can be fined between $1,000 and $5,000, and can be incarcerated for between one and three years (29).^2^ A history of incarceration is significantly related to recidivism (30) and future mental (31) and physical health problems (32). Incarceration can create or intensify financial hardships (33), affect one's ability to gain employment, secure a business or personal loan (34), work in certain industries, become politically involved, gain or keep a green card, and receive federal benefits such as student loans (35). Finally, having an incarcerated parent is associated with long long-term financial hardship, lower graduation rates, and poorer mental and physical health for children (36, 37).

## Legalization, the “green rush,” and race

Disappointingly, post legalization cannabis policies are being developed without the input of minority stakeholders (38, 39) or, to a large extent, the research community. This lack of input in combination with the fact that cannabis remains federally illegal and classified as a Schedule I substance has led to continued inequality. Barriers including high application fees and deposits on licenses (40), the inability to secure cannabis-related business loans (41), and rules preventing individuals with previous convictions from starting cannabis businesses (42) mean that the economic benefits of the so-called “Green Rush” are inequitably distributed, and a great deal of cannabis business has been concentrated among a small group: namely, wealthy white men.

For instance, the CEOs of the three cannabis companies with the greatest market share in the United States (Northwest Cannabis Solutions, Copperstate Farms LLC, and Mindful Medical) are all white, with a recent analysis reporting that over 81% of all cannabis business leadership is white (43). Additionally, with some notable exceptions (Solvay Pharmaceuticals, producers of Syndros for example), cannabis-based pharmaceuticals are overwhelmingly produced by white, male-led corporations, including Greenwich Biosciences (the producers of Epidiolex), Abbott Products (Marinol), GW Pharmaceuticals (Sativex), and Sanofi-Aventis (Acomplia). Thus, while this growing industry could have potential to be profitable for minority business owners, the legacy of racialized drug policy continues; while historical drug policy disproportionately targeted communities of color, legalization has disproportionately enriched wealthy white communities.

There have been attempts to shift cannabis industry policy in the direction of equity with varying degrees of success. For example, expungement of previous criminal records has been approached differently across states. Once cannabis was legalized in the state of Washington it took eight years to expunge the records of those convicted of cannabis charges before legalization, while in contrast, California made expungement a part of state legalization in 2016 (40). Expungement laws also vary widely by state, with some that provide expungement only to those convicted of possession, leaving those convicted of other cannabis-related crimes with their records intact. Expungement is also a lengthy and costly process that can take several years, typically requiring the engagement of legal counsel. These factors combined once again to disproportionately affect people of color who have not traditionally benefitted from the generational wealth and institutional policies that historically favor whites (44).

Some states have tried to develop equity-focused legalization policies around dispensary ownership, though these attempts are not without critiques. For example, New York has reserved a proportion of dispensary licenses for those directly affected by a marijuana-related incarceration (45), while Colorado's “accelerator program” is designed to help increase the number of cannabis business licenses for people of color (46). In Massachusetts, the Cannabis Social Equity Fund provides grants and loans to people of color to start cannabis businesses (Mass. General Laws c.94G § 14A|Mass.gov, 2022). Despite these efforts, however, communities of color continue to be excluded from most legal state markets, and many efforts continue to proceed without increased key stakeholder input from people of color. Given the fact that communities of color have been the most impacted by inequitable drug policy and enforcement historically, frustration and resentment continues to grow (47–51).

## Repercussions of cannabis commercialization for the research enterprise

At the same time, legalization has been largely motivated by economic forces while research on the risks or benefits of cannabis—hampered in large part by federal laws and regulations—is trailing largely behind usage trends and thus not informing policy as it should (52, 53). The field of cannabis research, while rapidly expanding, has yet to be integrated into the growth of industry and the development of state and federal policy at a level that is needed (54). As a result, many health care providers do not feel confident advising the public on the risks or benefits of cannabis, and often have a poor understanding of their state's cannabis laws (55–57).

It is also well documented that biomedical research has been conducted predominantly with participants who are white males of high socioeconomic status (e.g. 58), and cannabis research is no exception (59, 60). However, the lack of diversity in research is not only the result of the failure of researchers to adequately reach out to underrepresented communities (61–64) but is also impacted by a justifiable distrust of the research enterprise and the medical establishment by many minority groups (15). These issues of distrust are likely heightened for cannabis research, in particular, especially in the United States, though the subject is sorely understudied. Some recent work suggests that there is stigma and fear associated with medical cannabis use and worry about interactions with medical providers and law enforcement (65–68). Some other work has examined how fears and stigma may interact with individual marginalized identities related to gender (69, 70) or race (71, 72). Other work has begun to explore the relationship of the intersection of gender and race with cannabis-related stigma in the context of the cannabis industry (73). We were able to find one small study of stigma and cannabis research participation in general (not accounting for the race or ethnicity of respondents), that suggested willingness to participate in research was closely linked to levels of perceived stigma (74). While additional research is sorely needed in this area, we hypothesize that there are sizeable barriers to building a more inclusive cannabis research enterprise due to the historical reality of unequitable cannabis policy and enforcement, the stigma associated with cannabis use, and the broader distrust of the biomedical research establishment.

## Moving the field forward: policy and research recommendations

Clearly there have been and still are substantial and multifaceted barriers to diversity, equity, and inclusion in the cannabis research space. In the words of the American poet Amanda Gorman, “We will not march back to what was. We move to what shall be . . .” It is in that spirit that we move beyond the elucidation of the problems of the past to propose bold but feasible solutions for the future. Specifically, we propose below various ways that the field can move toward more equitable research within the domains of (1) research design, (2) support for underrepresented scientists in the field, and (3) state and federal cannabis policy. A summary of these suggestions can also be found in Table 1.

**Table 1.** Suggested actionable steps to improve cannabis research.

Follow best practices for ethical research design	Build mutually respectful community–research partnerships Diminish the distrust between minority groups and research Improve representation of minoritized groups as research participants Improve communication of research goals and share research results with the community Use qualitative methods to understand community values and goals and align the research goals with community goals
Support the entry, continuation, and retention of scientists from underrepresented groups	Collect demographic data on degree completion, degree switches, and dropping rates among students in order to improve STEM outcomes Offer more financial support to STEM students from underrepresented groups Make courses more inclusive to encourage participation of those with less research experience
Support more equitable cannabis policy at the state and federal level	Declassify cannabis as a Schedule I drug to improve access to the industry and help diminish stigma and fear for minority groups Support underrepresented groups in the cannabis industry Allow for federal bankruptcy protection Develop minority-focused policy and legislation Create a more inclusive industry Widen bank access to mitigate insurance risk

## Follow best practices for ethical research design

Though yet to be wholly embraced across biomedical research, the field of community engaged research has set forth both clear recommendations and actionable steps to improve representation and increase equity (75–81). These recommendations, including steps like improving communication of research goals and understanding of community values and goals, or investing in building mutually respectful community–research partnerships, are common sense approaches to begin to address distrust that has accumulated over decades. This approach could be especially fruitful for cannabis research given that many people may fear stigma or legal repercussions associated with participation that are unique compared to other areas of biomedical research. Our group has provided some actionable steps researchers could take to move toward more ethical design, such as developing participant registries, consistently communicating research results to communities, or using qualitative methods to align research goals with community goals before studies commence (60).

## Support the entry, continuation, and retention of scientists from underrepresented groups

Though we have evidence that the cannabis industry is exclusionary to people of color in terms of business ownership and pharmaceutical leadership, there are currently no statistics reporting on the demographics of cannabis-focused scientists in academia or in industry. Though sorely needed, this work would be challenging, given how interdisciplinary cannabis science is inclusive of fields like plant biology, genetics, agriculture, chemistry and biochemistry, medicine, and psychology and psychiatry to name a few. We do know, however, that STEM fields in general have problems recruiting, supporting, and retaining scientists from underrepresented groups; and even when these scientists are retained in research, there are substantial racial and gender pay gaps (82). Most of these problems arise well before the professional level, where women or students of color may not feel welcome to join studying certain topics (83), do not feel a sense of belonging in STEM educational disciplines (84, 85), or don’t feel supported to continue to graduation (86, 87).

The field would benefit from more research on the demographics of cannabis science, specifically, but also from concentrated efforts to improve STEM outcomes for underrepresented minority individuals. Some of the recommendations in the literature include requiring universities to collect demographic data on degree completion and switching/dropping rates among students, restructuring courses to be more welcoming to novices and inclusive of diverse students, increasing financial support and resources for STEM students from minoritized groups, and encouraging partnerships between institutions to share resources and programming (88, 89). One potential example of a productive partnership could be between new, specialized cannabis curriculums (e.g. 90) and POC STEM student support initiatives (e.g. https://mcnairscholars.com/).

## Support more equitable cannabis policy at the state and federal level

Most importantly for research, current legislative approaches severely limit researchers’ ability to study the health effects of cannabis. As the law stands, researchers cannot study legal market products on campuses and must rely on NIDA-approved suppliers with limited formulations that are not representative of the products available on the legal market (91). Additionally, it remains challenging for cannabis researchers to obtain permits from the FDA to study different cannabis formulations, and of course, these factors combine to negatively impact study participant recruitment (54).

Cannabis policy and legislative measures that are equity-focused are essential to both opening the research enterprise to minority groups and to decreasing stigma and fear around being “associated” with cannabis. This process should include addressing the current classification of cannabis under federal law as a Schedule I drug, which limits banking access, decreases insurance coverage options, and creates tax difficulties all under the specter of the risk of prosecution. For example, if cannabis were no longer a Schedule I drug, banks trying to conduct business within the cannabis industry would no longer be exposed to the risk of criminal charges or regulatory violations and could access FDIC deposit insurance for cannabis business funds (92). Once the cannabis industry shifts away from a cash-only business model, they would not rank as highly in terms of risk for insurers (93), and they would also be eligible to receive federal bankruptcy protection and apply federal tax deductions (94). These changes could then potentially open the industry to more small businesses, especially those led by people of color, and in turn help to decrease the stigma and fear around involvement in the cannabis industry. This would in turn distribute more of the profit from the cannabis industry to those communities most impacted by the criminalization of cannabis. Notably, state-run public funding schemes could also follow this pattern (e.g. California's Community Reinvestment Grants (CalCRG) program, 95).

## Conclusion

Substance use laws have been an arm of institutional power for hundreds of years and are often selectively enacted or enforced against minoritized communities. This has led to generations of people of color being disproportionately prosecuted and incarcerated for cannabis-related infractions, which in turn has contributed to inequity in the legal-market cannabis industry and in cannabis research. Confronting these power structures at the state and federal level with equity-focused legislation and policy, supporting the entry and retention of scientists of color into the field, engaging in more ethical research practices, and practicing intentionally inclusive recruitment of participants will help to move the field of cannabis research forward. Importantly though, these actions would also help ensure that the economic gains of the industry and the scientific benefits of research are equitably distributed.

## Footnotes

1 Of note, the Marijuana Tax Act of 1937 did not criminalize cannabis outright but imposed exorbitantly high “taxes” on anyone found growing, selling, or possessing cannabis (10).

2 Criminal history points are scored toward informing sentencing for federal crimes.
